# Combined Echo and Fluoroscopy-Guided Pulmonary Valvuloplasty in Neonates and Infants: Efficacy and Safety

**DOI:** 10.1007/s00246-021-02771-2

**Published:** 2021-11-28

**Authors:** Nicholas K. Brown, Nazia Husain, Jennifer Arzu, Sandhya R. Ramlogan, Alan W. Nugent, Paul Tannous

**Affiliations:** 1 Division of Cardiology, Department of Pediatrics, Ann & Robert H. Lurie Children’s Hospital of Chicago, Northwestern University Feinberg School of Medicine, 225 East Chicago Avenue, Box 21, Chicago, IL 60611 USA; 2 Department of Preventative Medicine, Northwestern University Feinberg School of Medicine, Chicago, IL USA

**Keywords:** Pulmonary valvuloplasty, Echocardiography guidance, Percutaneous intervention, Ionizing radiation, Contrast

## Abstract

Percutaneous balloon pulmonary valvuloplasty (PBPV) is the treatment of choice for isolated pulmonary valve stenosis. While this procedure is highly efficacious and has an excellent safety profile, as currently practiced, patients are obligatorily exposed to the secondary risks of ionizing radiation and contrast media. To mitigate these risks, we developed a protocol which utilized echo guidance for portions of the procedure which typically require fluoroscopy and/or angiography. Ten cases of echo-guided pulmonary valvuloplasty (EG-PBPV) for isolated pulmonary stenosis in children less than a year of age were compared to a historical cohort of nineteen standard cases using fluoroscopy/angiography alone, which demonstrated equivalent procedural outcomes and safety, while achieving a median reduction in radiation (total dose area product) and contrast load of 80% and 84%, respectively. Our early experience demonstrates that EG-PBPV in neonates and infants has results equivalent to standard valvuloplasty but with less radiation and contrast.

## Background

Percutaneous balloon pulmonary valvuloplasty (PBPV) is the treatment of choice for infants and neonates with isolated valvar pulmonary stenosis (PS) [1]. PBPV is a low-risk procedure, though the standard approach exposes the patient to ionizing radiation and iodinated contrast media [2]. No amount of radiation exposure is considered safe and children with congenital heart disease are particularly susceptible to the stochastic effects of ionizing radiation as they have immature organs, a longer anticipated lifespan following exposure, and are often exposed to a higher lifetime cumulative dose of radiation [3]. Considering these risks, there has been a global movement toward radiation reduction during cardiac catheterization procedures [4]. Furthermore, exposure to free iodide in contrast media can adversely affect thyroid function, causing transient but clinically significant hypothyroidism [5]. These exposure risks can be mitigated with the use of real-time, non-irradiating imaging modalities such as transthoracic and transesophageal echocardiography (TTE and TEE), which have been utilized to assist with device closure of shunt lesions such as atrial septal defects and patent ductus arteriosus, but have also played a role in limiting radiation exposure in extremely premature infants as well as pregnant and post-transplant patients [6–9].

Since 2019, the Interventional Cardiology Program at Lurie Children’s Hospital has used TTE guidance for PBPV in neonates and infants with isolated pulmonary valve stenosis. The initial experience using echocardiography-guided PBPV (EG-PBPV) for isolated pulmonary valve stenosis in patients less than 1 year of age is reported, testing the hypothesis that EG-PBPV has equivalent technical success and complication profile as compared to standard PBPV (S-PBPV), while reducing exposure to ionizing radiation and iodinated contrast media.

## Methods

### Developing the Echo-Guided Percutaneous Balloon Pulmonary Valvuloplasty Protocol

**Fig. 1:**
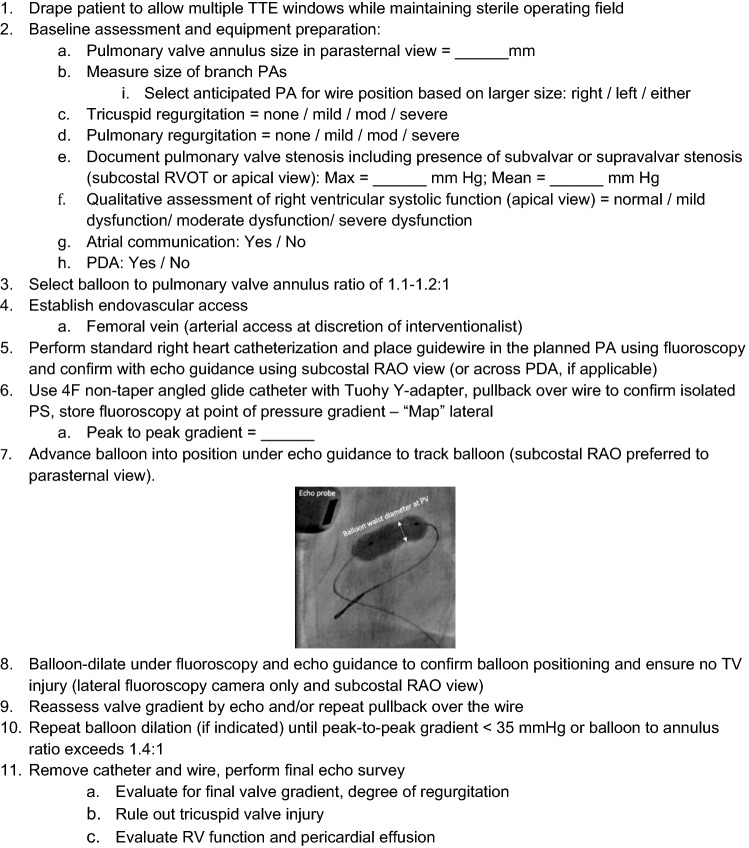
Patient preparation and protocol for echo-guided percutaneous balloon pulmonary valvuloplasty

A protocol for transthoracic EG-PBPV was created to mitigate contrast and radiation exposure (Fig. 1). Interventional and non-invasive imaging specialists performed a detailed review of our standard procedure and identified opportunities where echocardiography could be used as an alternative to fluoroscopy and/or angiography.

**Fig. 2:**
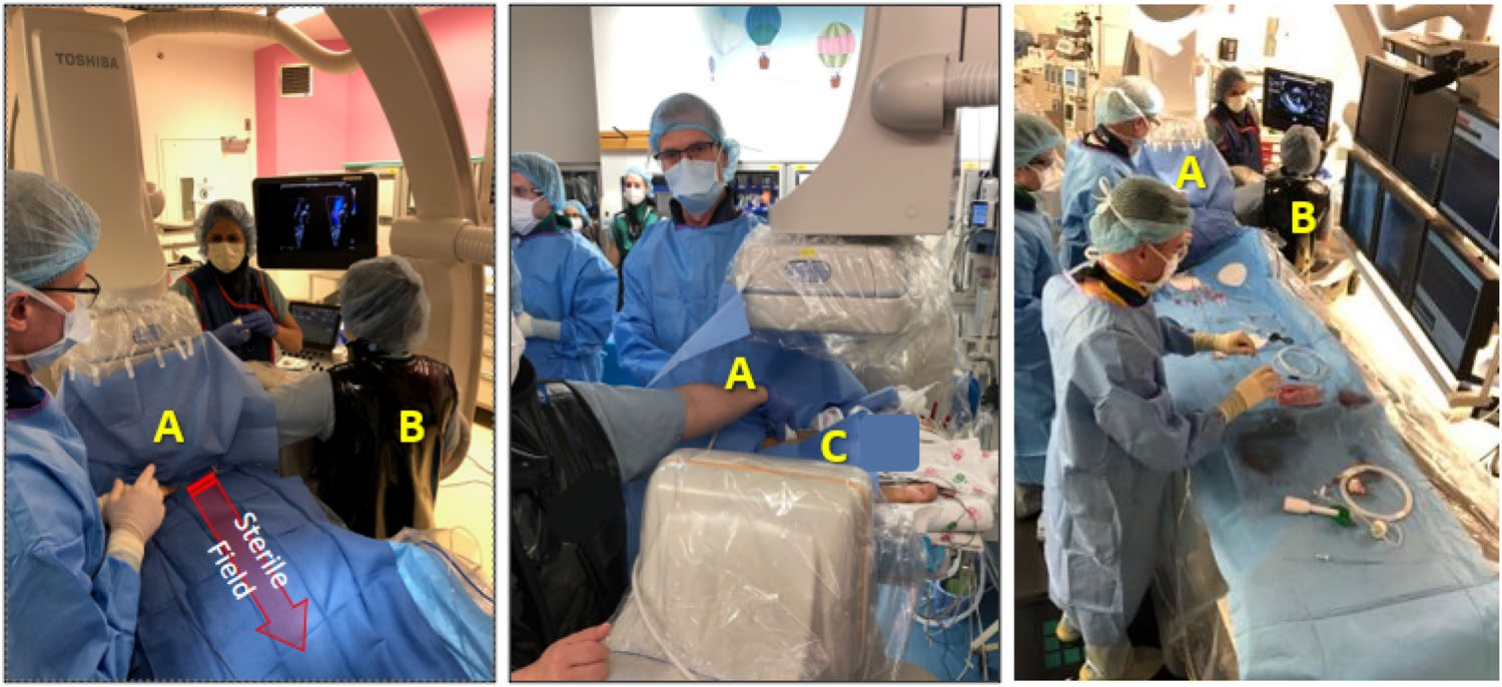
(A) Drape extended from patient abdomen to AP camera, dividing sterile and non-sterile fields. (B) Sonographer in position to allow comfortable access to patient while maintaining sterile field. (C) Drapes applied to allow subxiphoid, apical, and parasternal views

Key changes began with draping of the patient. Groins were prepped and draped in typical fashion, but drapes were secured to the lower abdomen using attached adhesive and taped to the anterior–posterior camera in order to create a sterile screen between the chest and groins and avoid contamination of the operating field (Fig. 2). This setup left the chest exposed to allow the imaging team access for subxiphoid, apical, and parasternal imaging windows. Adjustment of the location of the defibrillator patches, typically to the back, was also required to allow echocardiographic windows. The echocardiography machine was placed to the left of the patient table with the imaging screen positioned immediately lateral to the fluoroscopy cameras to allow visualization of both the echocardiography and fluoroscopy images by the primary echocardiographer and proceduralist. A subspecialized echocardiographer was present, with a trained technician as the primary operator. Before establishing endovascular access, a baseline echocardiographic survey was performed with attention to pulmonary valve annulus diameter from parasternal views, branch pulmonary artery size, tricuspid valve function, right ventricular systolic function, pericardial effusion, and importantly, for the presence of sub-valvar or supra-valvar obstruction.

Catheter manipulation during the right heart catheterization and guidewire positioning were done using standard fluoroscopy. The larger pulmonary artery from the echocardiographic survey was selected for wire position to ensure distal balloon accommodation during valve dilation. The balloon position across the pulmonary valve was confirmed using subcostal views and the dilation was performed with simultaneous 2D echocardiography and lateral camera fluoroscopy only with the sector collimated to ensure the sonographer’s hand was not in the beam path. A subcostal right anterior oblique (RAO) view by echocardiography was the most helpful for guiding catheter manipulation and balloon positioning as it provided a complete view of the relevant anatomy. After each dilation, various echocardiographic views were used to measure the residual valve gradient and degree of pulmonary regurgitation. We then used a 65-cm non-taper angled glide catheter (Terumo, Somerset, NJ) to perform pullback over the wire to document the residual peak-to-peak valve gradient. The procedure was considered complete once the peak-to-peak gradient was < 35 mmHg or the balloon-to-annulus ratio exceeded 1.4:1.

Upon completion, the catheter and wire were removed, and a final echocardiographic survey was performed to assess peak gradient and degree of pulmonary regurgitation in the absence of any instruments in situ*.* We also used this as an opportunity to evaluate for tricuspid valve injury or interval development of a pericardial effusion.

### Study Design

This was a single-center retrospective case–control study comparing EG-PBPV and S-PBPV procedures over a 3-year period from 12/2017 to 12/2020. From 9/2019, patients were considered EG-PBPV candidates if they were less than 12 months of age and presented with a pre-operative diagnosis of valvar pulmonary stenosis. All cases were performed with the patient under general anesthesia with the same biplane imaging system (Toshiba Infinix, Irving, CA) with similar fluoroscopy settings (default fluoroscopy frame rate 3 or 5 frames per second (FPS) and digital acquisition 15 FPS during the study period). Vascular access was established in a femoral vein in every case. Arterial access was obtained at the discretion of the interventionalist. EG-PBPV was performed with a Philips CVXi (Netherlands) echocardiographic system. Patients with atrial communications (ASD or PFO) were included, but those with more complex congenital heart defects or multilevel outflow tract obstruction were excluded. Demographic, echocardiographic, and procedural data included patient age, gender, weight, BSA, pre-operative peak instantaneous gradient by echocardiography, baseline peak-to-peak gradient by catheterization, residual peak-to-peak gradient post valvuloplasty by catheterization, total sheath time (min), contrast dose (ml/kg), fluoroscopy time (min), total dose area product or DAP (cGy cm^2^), and adverse events.

Primary outcomes were contrast dose (ml/kg), total dose area product (tDAP cGy cm^2^), and procedural success, which was defined as a residual valve gradient less than 35 mmHg on invasive hemodynamic assessment. Secondary outcomes were total sheath time, number of balloon sizes used, and number of adverse events.

Descriptive statistics were calculated for subject characteristics. Frequencies and percentages were reported for categorical variables, while median and interquartile ranges were displayed for continuous measures due to non-normal distributions. To assess statistically significant differences in selected characteristics (age, weight, body surface area, prostaglandin requirement, fluoroscopy time, and baseline catheterization gradient) and in outcomes (contrast dose, total radiation exposure, residual gradient, total sheath time, number of balloons), Wilcoxon rank-sum test or Fisher’s exact test were used. Statistical significance was assessed using a two-sided type I error rate of 0.05. All analyses were conducted using R (version 4.0.3) within RStudio (version 1.2.463).

## Results

Ten EG-PBPV cases were performed between 09/2019 and 12/2020 and nineteen underwent S-PBPV between 12/2017 and 10/2019. This was due to a change in clinical practice with the goal of incorporating echocardiography guidance in the interventional laboratory. All EG-PBPV studies were performed by three echocardiographers and two interventionalists. There were no significant differences in age, weight, or BSA between the two groups (Table 1). Two patients (20%) in the EG-PBPV group and six patients (32%) in the S-PBPV group presented as critical PS (with need for prostaglandin infusion). There was no difference in baseline invasive peak-to-peak pulmonary valve gradients (43.5 mmHg EG-PBPV versus 52.0 mmHg S-PBPV, *p* = 0.425).

**Table 1:** Baseline characteristics by echo-guided status

		Echo-guided status		
	Overall^a^ (*n* = 19)	No^a^ (*n* = 19)	Yes^a^ (*n* = 10)	*p* value^b^
Age (days)	36.0 (5.0–135.0)	48.0 (4.5–136.0)	32.5 (23.5–96.0)	0.872
Weight (kg)	3.8 (3.2–5.9)	3.7 (3.0–6.0)	4.2 (3.4–4.8)	0.614
BSA (m^2^)	0.23 (0.20–0.30)	0.22 (0.20–0.31)	0.24 (0.21–0.27)	0.713
PGE	8 (28%)	6 (32%)	2 (20%)	
Baseline cath gradient (mmHg)	46.0 (40.2–56.0)	43.5 (31.8–53.2)	52.0 (42.8–56.0)	0.425

*PGE* prostaglandin requirement

^a^Median (IQR), *n* (%)

^b^Wilcoxon rank-sum test

**Fig. 3:**
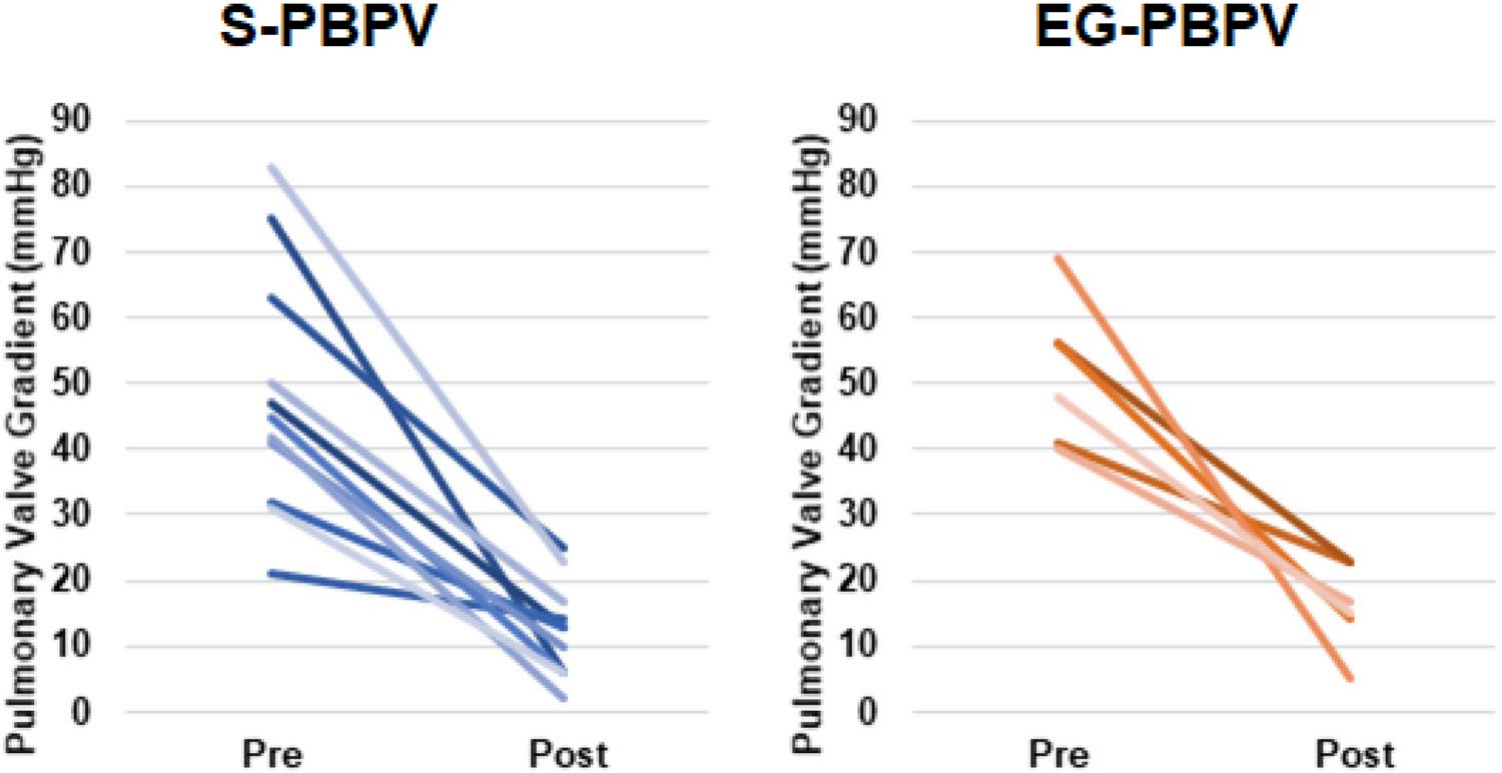
Pre- and Post-intervention pulmonary valve gradients between S-PBPV and EG-PBPV cohorts show equivalent success measured as catheter-derived peak-to-peak valve gradient before and after the procedure (created in Microsoft excel)

EG-PBPV achieved outcomes similar to S-PBPV with significant reductions in patient exposure to ionizing radiation and contrast media (Table 2; Fig. 3). There was no significant difference in the residual peak-to-peak gradient between each group (13.0 mmHg with EG-PBPV versus 14.5 with S-PBPV, *p* = 0.713). Total dose area product (tDAP) in the EG-PBPV was reduced by 80% compared to S-PBPV (33.8 versus 167.4 cGY cm^2^, *p* < 0.001) and contrast load was reduced by 84% (0.8 versus 5.0 ml/kg, *p* = 0.003). There was no significant difference in fluoroscopy time (10.8 versus 8.3 min, *p* = 0.136).

**Table 2:** Comparison of results between standard and echo-guided PBPV

	S-PBPV^a^ (*n* = 19)	EG-PBPV^a^ (*n* = 10)	*p* value^b^
Residual cath gradient (mmHg)	13.0 (6.0–19.0)	14.5 (8.5–20.8)	0.713
Contrast dose (ml/kg)	5.0 (4.5–8.5)	0.8 (0.0–3.5)	0.003
tDAP (cGy cm^2^)	167.4 (82.2–266.8)	33.8 (25.9–60.7)	< 0.001
Fluoroscopy time (min)	10.8 (7.7–14.2)	8.3 (7.4–9.8)	0.136
# adverse events reported (*n*)	0	0	1.00
> 1 balloon required	4 (21%)	5 (50%)	0.205
Maximum balloon-to-annulus ratio	1.2 (1.1–1.2)	1.2 (1.1–1.2)	0.645
Total sheath time (min)	50.0 (33.5–56.0)	36.5 (30.0–45.8)	0.241

*tDAP* dose area product (total radiation exposure), *cGy* centigray

^a^Median (IQR), *n* (%)

^b^Wilcoxon rank-sum test, Fisher’s exact test

**Fig. 4:**
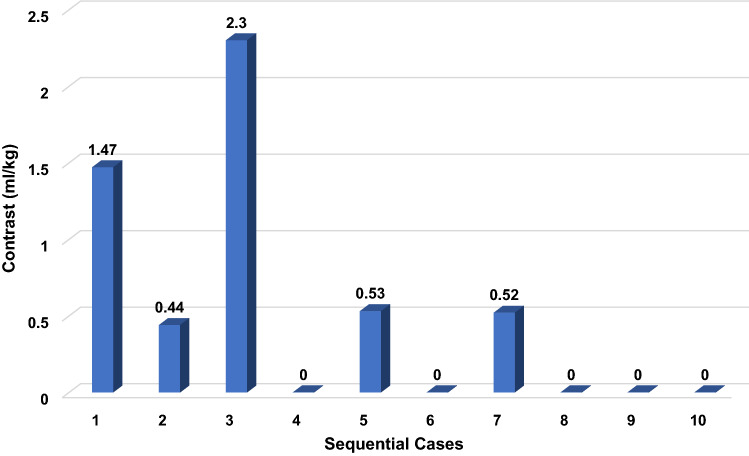
Technical proficiency improved over time with lower use of contrast with sequential EG-PBPV cases which are numbered 1–10 from first to most recent case (created in Microsoft excel)

**Fig. 5:**
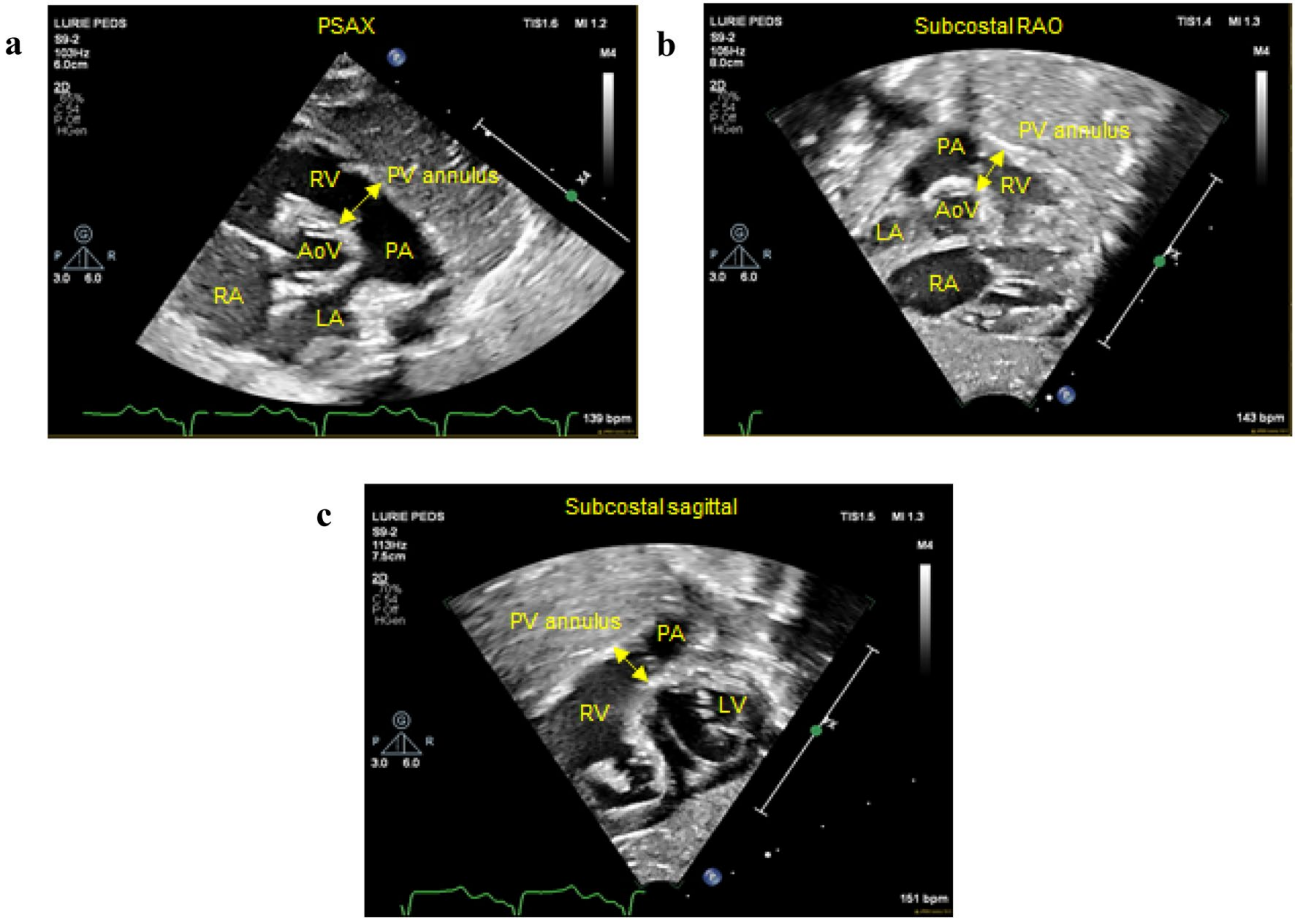
Key echocardiographic views demonstrating measurement of pulmonary valve annulus: **a** PSAX view, **b** subcostal RAO view, and **c** subcostal sagittal view. *PSAX* parasternal short axis, *RAO* right anterior oblique, *RA* right atrium, *TV* tricuspid valve, *RV* right ventricle, *RVOT* right ventricular outflow tract, *PV* pulmonary valve, *PA* pulmonary artery, *LA* left atrium, *LV* left ventricle, *AoV* aortic valve

**Fig. 6:**
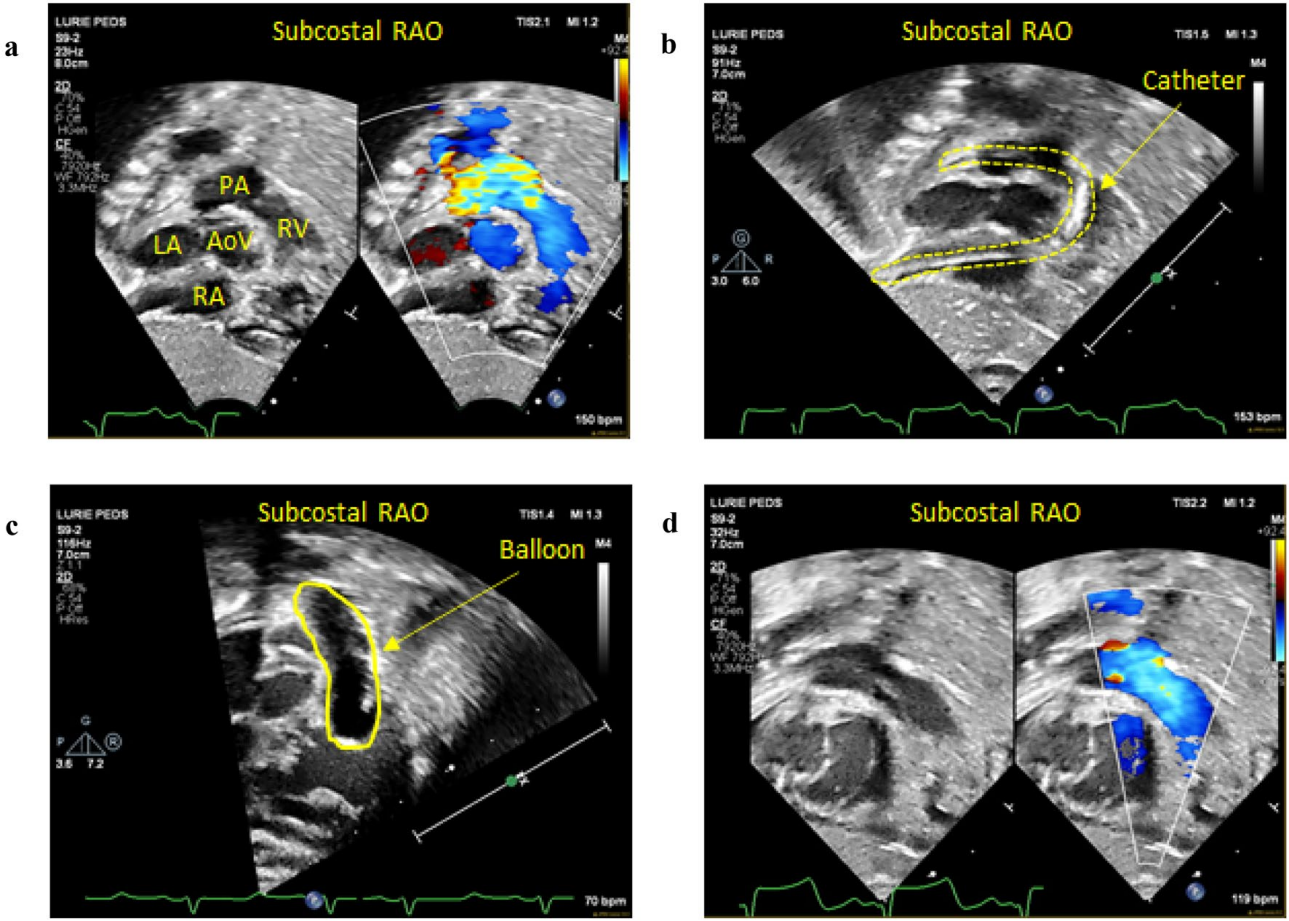
Key intra-procedure echocardiographic findings during EG-PBPV. **a** Baseline assessment showing increased flow velocity by color Doppler starting at the level of the pulmonary valve without sub- or supravalvular pulmonary stenosis. **b** Subcostal RAO view showing catheter coursing from IVC across TV through RVOT and into RPA (highlighted with dashed lines). **c** Subcostal RAO view showing balloon inflated across pulmonary valve (outlined in yellow). Balloon is not impinging on TV. **d** Following balloon valvuloplasty, the flow acceleration across the pulmonary valve has resolved. *RAO* right anterior oblique, *RA* right atrium, *RV* right ventricle, *PA* pulmonary artery, *LA* left atrium, *AoV* aortic valve

Reviewing cases in a sequential manner, we find evidence of progressively decreased contrast load as our team became more facile with EG-PBPV, with five of the last six cases performed entirely contrast free (Fig. 4). As angiograms to measure the pulmonary valve annulus are typically performed with high-energy cine acquisition, there is a direct correlation between contrast dose and total radiation exposure which were both significantly reduced by measuring the pulmonary valve using echocardiography (Figs. 5,6). With echo guidance, total sheath time decreased (50 min S-BPVP versus 36.5 min EG-BPVP), but this difference was not statistically significant (*p* = 0.241). Of note, there were no differences in the number of balloons used, final balloon-to-annulus ratio, fluoroscopy time, or the number of adverse events.

## Discussion

Highly efficacious methods for percutaneous balloon pulmonary valvuloplasty have already been established, and this procedure has a well-documented excellent safety profile. Here, we report how the addition of echocardiography guidance with a multi-disciplinary team approach enabled achievement of identical outcomes in terms of efficacy and safety while significantly reducing the secondary risks related to ionizing radiation and contrast load in neonates and infants.

With advances in non-invasive imaging technology, there have been significant opportunities to form collaborations between imaging and interventional teams. This has the potential to improve clinical care by utilizing the strengths of each modality to reduce the risks associated with caring for pediatric patients with congenital heart disease. Although PBPV is a low-risk procedure, we were able to develop a protocol which significantly lowered the amount of ionizing radiation and contrast required to achieve technical success in a population composed primarily of neonates and infants. Identical outcomes were achieved without an increase in significant adverse events or total case time. It was anticipated that increasing the number of people involved in decision making would prolong the procedure, but the opposite was found with a non-significant reduction in sheath time (which is a surrogate for procedure time).

The technical proficiency also improved over time with evolution of communication and ideal echocardiography views. Echocardiography guidance was used for portions of the procedure that have historically required radiation/contrast exposure such as advancing the balloon catheter into position, measuring the pulmonary valve annulus, and monitoring the tricuspid valve during balloon inflation. Using 2D echocardiography to determine pulmonary valve annulus size allowed us to minimize and eventually eliminate the contrast load in many cases, while also avoiding high-energy cine imaging. Avoiding cine imaging was the primary reason for significantly decreased DAP in the echo-guided group even though fluoroscopy times were similar. This study reports the first ten EG-PBPV cases including our learning curve, thus with increasing experience of the echocardiography and interventional teams, efficiencies will only continue to improve and perhaps procedure time reduction may also become significant.

This institution, like others, has undergone changes in an effort to reduce radiation over the last several years [10]. The historical cohort of S-PBPV had a median DAP of 167 cGY cm^2^ that compares favorably to benchmark radiation data for PBPV intervention under age 1 year that reported a median DAP of 249 cGY cm^2^ [4]. While improvements in technology with modern systems can reduce radiation further [11], all catheterization laboratories have by now already maximized procedural strategies to lower radiation (frame rate, collimation, stored fluoroscopy). Thus, further reductions in radiation will require adoption of real-time imaging that does not use ionizing radiation such as echocardiography or magnetic resonance imaging [12].

TTE and TEE guidance in the interventional suite has historically and more commonly been used for percutaneous ASD closure. Jone et al. reported that the addition of 3D TEE to interventional ASD closure procedures led to a significant reduction in radiation exposure [6]. Ackermann et al. used exclusive TEE guidance without fluoroscopy for ASD closure in a larger cohort of pediatric patients which showed the feasibility of this approach although fluoroscopy is often needed for more complicated ASD closures and smaller patients where a 3D imaging probe is unavailable [13]. Proof that this strategy of exclusive echocardiography guidance could be extended to pediatric pulmonary valvuloplasty was reported by Wang et al. [14]. These patients were an older cohort (mean age 8.7 ± 3.7 years) than the present report of neonates and young infants, documenting that EG-PBPV can be safely performed even in higher risk neonates with critical pulmonary stenosis. This same group has also performed exclusively echo-guided percutaneous balloon aortic valvuloplasty (again in an older cohort with a mean age of 18.38 ± 15.88 years), although this procedure carries a significantly higher risk when performed in the neonatal period [15].

In considering the feasibility of measuring the pulmonary valve exclusively by echocardiography, Dev et al. revealed a good correlation between echocardiographic and angiographic pulmonary valve measurements [16]. This study was reproduced by Chubb et al. who similarly reported good correlation between contemporary measurements, albeit with relatively wide limits of agreement [17]. As we gradually phased out measuring the pulmonary valve annulus by angiography with contrast and high-energy cine imaging, we did not perform a comparison of echocardiographic and angiographic measurements. In addition, there were no significant differences in the number of balloons used in either cohort, highlighting the reliability of echocardiographic measurements in balloon selection during EG-PBPV. With technological advancements in transthoracic echocardiography and the advent of pediatric 3D probes, measurement of the pulmonary valve in orthogonal planes may further assist in accurate selection of balloon sizes during EG-PBPV.

There may be other pediatric populations that benefit from more routine use of echocardiography in the catheterization lab such as aortic valvuloplasty, premature infants requiring PDA closure, and post-transplant patients who require frequent exposure to ionizing radiation as a part of routine surveillance. Chandrasekhar et al. revealed that echo-guided endomyocardial biopsy is safe and led to less radiation exposure without extending the procedure time [7]. Many of these transplant patients have a history of congenital heart disease and have already been exposed to a significant amount of radiation pre-transplant [3]. Application of novel strategies like the use of real-time echocardiography for catheter-guidance may lower their future radiation exposure and associated risks.

Our study was limited by the fact that this was a single-center retrospective study reporting our initial experience. There is potential inter-operator variability among catheterization and imaging personnel which could affect outcomes as well as a possible era effect given that echo-guided cases were performed at a different time period compared to our historical cohort. We expect that resource utilization within a pediatric cardiology group may be affected by this change in practice as it requires an echocardiographer to be present for the entirety of the procedure in the catheterization suite. Although a reduction in procedure time may be possible with this approach, adding an echocardiographic survey prior to the start of the case could potentially affect total case/anesthesia time. Due to a small initial cohort size, our study was not powered to detect operator differences. Lastly, other radiation variables could not be as easily quantified (if at all) such as collimation and image intensifier distances.

## Conclusions

Our early experience demonstrates that EG-PBPV in neonates and infants has results equivalent to standard valvuloplasty but with less radiation and contrast. Further improvements are anticipated with increasing experience as protocol, communication, and team dynamics are solidified resulting in significantly less contrast, fluoroscopy, and perhaps even procedure time.

## Data Availability

Yes.
